# Pharmacological Activation of Nrf2 by Rosolic Acid Attenuates Endoplasmic Reticulum Stress in Endothelial Cells

**DOI:** 10.1155/2021/2732435

**Published:** 2021-04-08

**Authors:** Karan Naresh Amin, Palanisamy Rajagru, Koustav Sarkar, M. R. Ganesh, Takayoshi Suzuki, Daoud Ali, Ramkumar Kunka Mohanram

**Affiliations:** ^1^SRM Research Institute and Department of Biotechnology, School of Bioengineering, SRM Institute of Science and Technology, Kattankulathur, 603 203 Tamilnadu, India; ^2^Department of Life Sciences, Central University of Tamil Nadu, Tiruvarur 610005, India; ^3^Interdisciplinary Institute of Indian System of Medicine, SRM Institute of Science and Technology, Kattankulathur, 603 203 Tamilnadu, India; ^4^Division Cellular and Gene Therapy Products, National Institute of Health Sciences, Setagaya-Ku, Tokyo, Japan; ^5^Department of Zoology, College of Science King Saud University, P.O. Box 2455, Riyadh 11451, Saudi Arabia

## Abstract

Endoplasmic reticulum (ER) plays a key role in the folding, modification, and trafficking of proteins. When the homeostasis of the ER is disturbed, un/misfolded proteins accumulate in the ER which leads to ER stress. Sustained ER stress results in apoptosis, which is associated with various diseases. Nuclear factor erythroid 2-related factor 2 (Nrf2) is a major transcription factor in redox homeostasis by regulating various genes associated with detoxification and cell-protective mechanisms. We found that Rosolic acid (RA) treatment dose-dependently activates Nrf2 in endothelial cells using the enzyme fragment complementation assay. The cytoprotective role of RA against ER stress-induced endothelial apoptosis and its molecular mechanism was explored in the present study. The Nrf2 and its target genes, as well as ER stress marker expressions, were measured by qPCR in ER stress-exposed endothelial cells. The contribution of Nrf2 in RA-mediated defense mechanism in endothelial cells was established by knockout studies using Nrf2-CRISPR/Cas9. The treatment with RA to ER stress-induced endothelial cells exhibited activation of Nrf2, as demonstrated by Nrf2 translocation and reduction of ER stress markers. We found that the Nrf2 knockout sensitized the endothelial cells against ER stress, and further, RA failed to mediate its cytoprotective effect. Proteomic studies using LC-MS/MS revealed that among the 1370 proteins detected, we found 296 differentially regulated proteins in ER stress-induced endothelial cells, and RA administration ameliorated 71 proteins towards the control levels. Of note, the ER stress in endothelial cells was attenuated by the treatment with the RA, suggesting the role of the Nrf2 activator in the pathological conditions of ER stress-associated diseases.

## 1. Introduction

The endothelium is the main component of the barricade for transporting many important biological compounds from the blood to the inner layer of cells. It is responsible for maintaining the vasculature by releasing various vasodilatory or vasoconstrictive factors like NO, endothelin-1, endothelium-derived hyperpolarizing factor, prostacyclin, and thromboxane [1]. Endothelial cells form a key component of the inflammatory and immune response and are the first cells to be activated during inflammation [2]. Endothelial apoptosis is a causative factor for diabetes and its vascular complications [3]. Understanding the transitions in apoptotic pathways may allow an improved understanding of the pathogenesis of disease and in the development of novel therapies [4, 5]. The currently available research highlights endothelial apoptosis as an initial event in the progression of certain diseases, including diabetes [6, 7].

The perturbations to the endothelial cell metabolic pathways lead to oxidative stress, endoplasmic reticulum (ER) stress, and inflammatory processes, which are the important contributors to endothelial apoptosis. ER stress has increased more attention as a crucial factor connecting and congregating molecular relationships among oxidative stress, endothelial cell dysfunction, and insulin resistance and, therefore, is considered an auspicious drug target for controlling diabetes and cardiovascular problems. Increasing evidence identifies the cross talk between oxidative stress and ER stress in the pathogenesis of diabetes and its complications [8, 9].

Numerous therapeutic strategies have been developed to combat ER stress-induced endothelial cell dysfunction [10–12]. Nrf2 is a major transcription factor in cellular defense, which acts as the chief watchdog of reduction-oxidation status and detoxification. Nrf2 is generally found in the cytoplasm under physiological conditions and connected with its negative regulator, Kelch-like ECH-associated protein1 (Keap1) [13, 14]. When the cells encounter oxidative stress or electrophiles, Nrf2 detaches from the Nrf2-Keap1 complex and translocates to the nucleus that activates the gene expression of antioxidant responsive element (ARE) to sustain cellular redox homeostasis [15]. In recent years, activation of Nrf2 by small molecules have been demonstrated as a new promising therapeutic approach for counteracting endothelial apoptosis [16–19]. It is interesting to note that Nrf2 is reported to associate with the UPR sensor called pancreatic endoplasmic reticulum kinase [20].

We have developed a high-throughput screening system based on luciferase complementation to screen Nrf2 activators and demonstrated its role against oxidative and cytokine stress in pancreatic beta cells through Nrf2 activation [21–25]. Few Nrf2 activators showed promising results against hyperglycemia-induced endothelial dysfunction (ED) [16]. Earlier, we reported quercetin, a potent Nrf2 activator that showed a cytoprotective effect on endothelial cells against ER stress-induced ED [26]. We have established a co-culture system to examine the cross talk between pancreatic beta cells and endothelial cells under ER stress and validated with a recognized ER stress regulator, quercetin [27].

Rosolic acid (RA), an important polyphenol extracted from *Plantago asiatica* L., has been demonstrated to increase the levels of Heme oxygenase-1 (HO-1) in endothelial cells [28]. The role of RA on ER stress-induced ED and its effect as a therapeutic agent are yet to be revealed. Hence, this study investigated the effects of RA against ER stress-induced toxicity in endothelial cells. Further, the role of Nrf2 on RA-mediated cytoprotection was also investigated using Nrf2 knockout endothelial cells by CRISPR/Cas9. To offer better understanding of the underlying molecular mechanism, we have carried out a proteomic study on the RA-treated ER stress-induced endothelial cells using electrospray ionization tandem mass spectrometry (LC-MS/MS).

## 2. Materials and Methods

### 2.1. Cell Culture

The immortalized human umbilical vein endothelial hybrid cell line, EA.hy926 cells, was maintained in DMEM medium (Clonetics; Lonza Ltd, Basel, Switzerland) along with Fetal bovine serum (FBS, 10%) (*v*/*v*) (HyClone, Logan, UT, USA), penicillin (100 U/ml), and streptomycin (100 *μ*g/ml). The cells were kept in a humidified 5% CO_2_ atmosphere at 37°C. Immortalized cell lines offer significant logistical advantages over primary cells when used for *in vitro* studies.

### 2.2. ER Stress Induction

ER stress was induced in the EA.hy926 cells by exposing the cells to thapsigargin (TPG) (Sigma-Aldrich, USA) at a dose of 2 *μ*M for two hours [29]. TPG inhibits sarcoplasmic/endoplasmic reticulum Ca^2+^-ATPase, leading to a depletion of ER calcium storage, resulting in the accumulation of unfolded proteins and inducer of cell death [30].

### 2.3. Assay of Cell Viability

The cell viability was measured using the MTT assay [31]. In brief, EA.hy926 cells (1x10^4^ cells/well) were cultured in a 96-well plate for 24 h at 37°C and then pretreated with RA (0-100 *μ*M) for the next 24 h. After incubation, the medium was replaced by MTT solution (5 mg/ml) and kept in a humidified 5% CO_2_ atmosphere for 4 h at 37°C. 100 *μ*l of DMSO was added to all the wells and mixed thoroughly and read at 570 nm by a microplate reader (TECAN, Switzerland).

To study the effect of RA on ER stress-induced cell death, the cells were pretreated with RA (0-100 *μ*M) for at least 24 h trailed by TPG (2 *μ*M) for 2 h. The assay of MTT was then carried out as described above.

### 2.4. Quantitative Real-Time PCR (qRT-PCR)

The molecular ER stress marker profiles, protein kinase R- (PKR-) like endoplasmic reticulum kinase (PERK), glucose-regulated protein (GRP) 78, C/ERB homologous protein (CHOP), activating transcription factor- (ATF-) 6, and Nrf2-regulated genes, including NAD(P)H quinone-oxidoreductase-1 (NQO1), heme oxygenase-1 (HO-1), catalase (CAT), and glutathione peroxidase (GPx), were performed using qRT-PCR (Bio-Rad, USA). The primers for the targeted genes were obtained from the genome DNA sequence database and NCBI human mRNA, and primers were designed using the primer3 plus, a free online bioinformatics tool, which is enumerated in Table 1. The cells were treated with different concentrations of RA for 24 h followed by thapsigargin (2 *μ*M) for 2 h. The mRNA isolation was performed using the mRNA isolation kit (Qiagen, Germany), followed by the cDNA conversion using a commercially available kit (Qiagen, Germany) as per instructions provided by the manufacturer. The qRT-PCR was achieved using SYBR® Premix Ex Taq™ II (TaKaRa, Japan) as per the instructions provided by the manufacturer. The housekeeping gene Glyceraldehyde-3-phosphate dehydrogenase (GAPDH) was used as the internal control for normalization, and the results are represented as relative fold change.

### 2.5. ARE-Luciferase Reporter Gene Assay

To confirm the Nrf2 activation potential, ARE promoter Responsive Luciferase Reporters such as GST-ARE-Luc and hNQO1-ARE-Luc reporter gene constructs were used in endothelial cells. Briefly, EA.hy926 cells were seeded and transfected with an ARE-Luc construct (500 ng/well) with the help of Lipofectamine 2000. After 24 h, the transfected cells were administered with different amounts of RA (0-20 *μ*M) and kept incubated for 8 h, and then the cells were assayed for luciferase activity by a luminometer (Promega, Madison, WI, USA). The luciferase activity was expressed in terms of relative fold variation compared to control cells.

### 2.6. Measurement of Intracellular Reactive Oxygen Species (ROS) Formation

2',7'-Dichlorofluorescin diacetate (DCFDA) (Sigma-Aldrich, USA) was used to measure the intracellular peroxides to assess the protective effect of RA against ROS formation in ER stress-induced endothelial cells. In brief, the endothelial cells were pretreated with RA (5 and 10 *μ*M) for 24 h followed by exposure with 2 *μ*M TPG for 2 h. Then, the cells were treated with DCFDA solution (25 *μ*M) for 15 min, and the fluorescence was measured using flow cytometer (BD Biosciences, CA, USA) at 502 nm excitation and 550 nm emission wavelength.

### 2.7. Apoptosis Detection Using Annexin-V Labeling Assay by Flow Cytometry

Annexin-V-FITC was used to measure the apoptotic population percentage to evaluate the protective effect of RA against ER stress-induced endothelial cells. Briefly, the cells were harvested and centrifuged for 3 minutes at 1500 rpm. The cells were then stained with Annexin-V-FITC and determined using flow cytometer (BD Biosciences, CA, USA).

### 2.8. CRISPR/Cas9-Mediated Knockout of Nrf2 in Human Endothelial Cells

To confirm the role of RA on Nrf2-mediated protection, Nrf2-KO endothelial cells were generated using the CRISPR/Cas9 gene-editing tool. EA.hy926 cells were transfected with Nrf2-CRISPR/Cas9-GFP plasmid (sc-400017, Santa Cruz, USA). GFP^+^ cell expression was screened using FACS. Western blot analysis for Nrf2-expression was performed to confirm successful Nrf2-depletion. Further, the Nrf2 role in RA which facilitated the protection of endothelial cells against ER stress was assessed using Nrf2 KO cells by assessing the expression of ER stress markers and cell viability.

### 2.9. Immunoblot Analysis

The cytosolic and nuclear extracts were prepared by using the NE-PER™ Nuclear and Cytoplasmic Extraction kit (Thermo Scientific, USA). Further, the whole-cell protein lysate from the cells was prepared using RIPA buffer, and the concentration of protein was measured using Bradford reagent (Bio-Rad, PA, USA). Equal quantities of proteins were used in SDS-PAGE and transferred to a nitrocellulose membrane. Then, they subjected to blocking with BSA (5%), followed by immunoblotting using respective primary antibodies, *β*-actin (Abcam, USA), Nrf2 (Santa Cruz, USA), GRP78 (Santa Cruz, USA), Lamin B1 (Santa Cruz, USA), CHOP (Santa Cruz, USA), and corresponding secondary antibody, anti-rabbit IgG, and HRP-linked antibody. The expression levels were detected by the enhanced chemiluminescence using (ECL) kit (Bio-Rad, PA, USA) and the ECL western blotting detection reagent, and the protein bands were captured using the ChemiDoc system (GBOX, Syngene, UK).

### 2.10. Enzyme Activities

The superoxide dismutase (SOD) activity was measured based on the nitro blue tetrazolium (NBT) reduction assay [32]. Catalase (CAT), and glutathione peroxidase (GPx) activities were determined by measuring the amount of the substrate consumed by colorimetrically [33].

### 2.11. Proteomic Investigations Performed by Nano LC-MS/MS Analysis

The molecular mechanism of RA-mediated protection in ER stress-induced endothelial cells was studied by the LC-MS/MS analysis. The protein digests were determined by nanoflow HPLC–electrospray tandem mass spectrometry (LC-MS/MS). The comprehensive methodology is provided in the supplementary material (available here).

### 2.12. Statistical Analysis

All experimental studies were completely achieved in a randomized design. One-way ANOVA followed by Tukey's post hoc test was subjected using the SPSS version 17.0 software (SPSS Inc., Chicago, IL, USA). All outcomes were reported as mean ± SD of at least independent tests of triplicates and *P* ≤ 0.05 was measured as statistically significant.

## 3. Results

### 3.1. Cytoprotective Effect of RA on TPG-Stimulated ER Stress in Endothelial Cells

**Figure 1:**
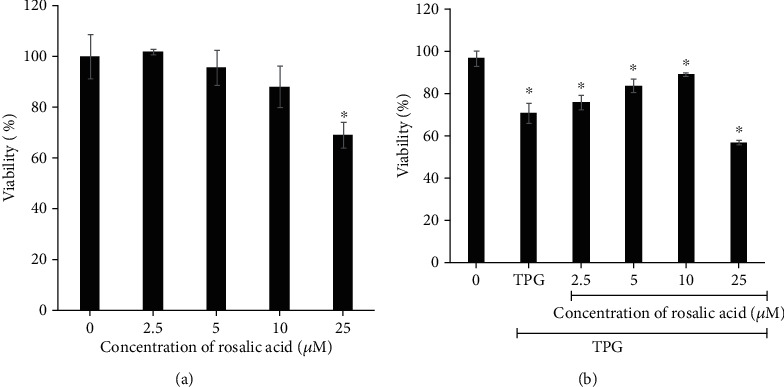
Effect of Rosolic acid on ER stress-induced toxicity in EA.hy926 cells. (a) Cytotoxicity of Rosolic acid on EA.hy926 cells as assessed by MTT assay. (b) Cytoprotective effect of Rosolic acid on EA.hy926 cells against thapsigargin-induced toxicity as assessed by MTT assay. Data are represented as mean ± SD of three independent experiments. ∗Significant compared with the control group; *P* < 0.05 determined by one-way ANOVA followed by Tukey's post hoc test (0, control; TPG, thapsigargin; 2.5, 5, 10, and 25, concentrations of Rosolic acid in *μ*M).

To evaluate the cytotoxic activity of RA in endothelial cells, cells were exposed to different concentrations of RA for 24 h, and viable cells were determined by the MTT assay. The outcomes are presented in Figure 1(a). The cytoprotective effect of RA against TPG-induced apoptosis in endothelial cells was accessed using the MTT assay. Endothelial cells treated with TPG at 2 *μ*M for 2 h showed a 35% reduction in cell viability. However, the cells pretreated with different concentrations of RA (2.5-25 *μ*M) showed a dose-dependent increase in cell viability, and the protective effect was found to be maximum at 5 and 10 *μ*M RA concentrations (Figure 1(b)), and at 25 *μ*M, it was toxic to the cells.

### 3.2. Nrf2 Activation Potential of RA in Endothelial Cells

**Figure 2:**
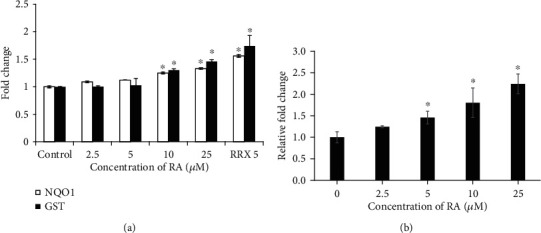
Nrf2 activation potential of Rosolic acid as assessed by ARE-Luciferase Reporter Assay. (a) HEK293T cells were transfected with either ARE-hNQO1/ARE-GST luciferase vector. Transfected cells were treated with Rosolic acid (0, 2, 5, 10, and 20 *μ*M) for 8 h. A known Nrf2 activator, RRx, was used as a reference control. Luciferase activity was measured as described in methods and expressed as fold induction relative to values obtained from control cells. Photon flux data were normalized to total protein. Effect of Rosolic acid on Nrf2 expression in EA.hy926 cells (b). Data are represented as mean ± SD of three independent experiments. ∗Significant compared with the control group; *P* < 0.05 determined by one-way ANOVA followed by Tukey's post hoc test.

To determine the Nrf2 activation by RA, its binding potential to nuclear ARE and activation of its downstream regulatory genes, reporter gene constructs, and their cell-based assays, GST-ARE-Luc and hNQO1-ARE-Luc were employed. Various concentrations of RA treated on endothelial cells, which were later harvested and measured the luciferase activity. A dose-dependent elevated reporter luciferase signals of GST and NQO1 were observed in RA-treated cells, presented in Figure 2(a). Furthermore, dose-dependent elevated levels of Nrf2 in RA-treated endothelial cells are also noticed in Figure 2(b). Based on the outcome, the study confirms the potential role of RA on the activation of Nrf2 in endothelial cells.

### 3.3. Efficacy of RA on Nrf2 Activation and Translocation in ER Stress-Stimulated Endothelial Cells

**Figure 3:**
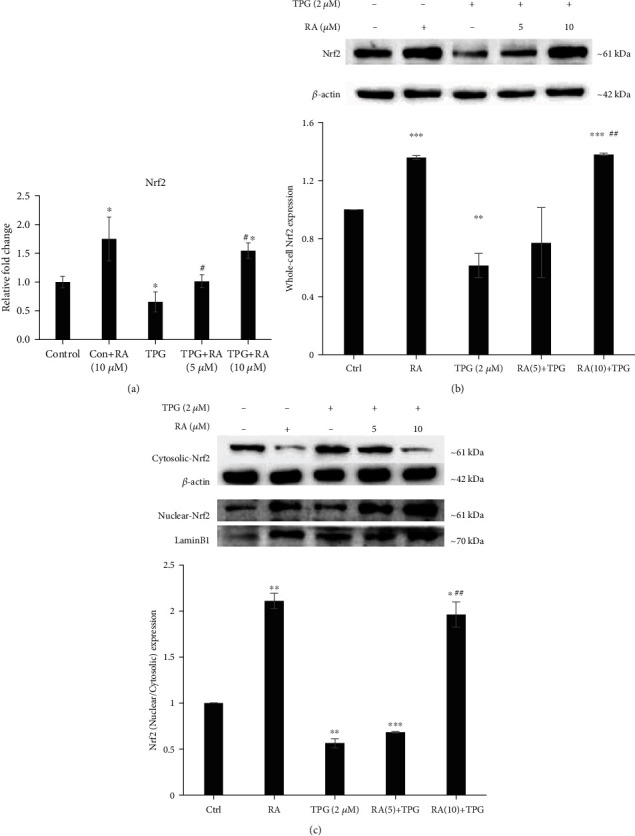
Effect of Rosolic acid on level of Nrf2 gene expression (a) by qRT-PCR analysis and protein level (b) by western blotting in ER stress-induced EA.hy926 cells. (c) The effect of Rosolic acid on Nrf2 translocation from cytosol to the nucleus by western blotting in EA.hy926 cells under ER stress. Data are represented as mean ± SD of three independent experiments. ∗Significant compared with the control group. ^#^Significant compared with TPG group; *P* < 0.05 determined by one-way ANOVA followed by Tukey's post hoc test (0, control; TPG, thapsigargin; 2.5, 5, 10, and 25, concentrations of Rosolic acid in *μ*M).

To study the role of RA on Nrf2-mediated protection of endothelial cells against ER stress, we evaluated the impact of RA on the Nrf2 expression and its downstream target genes in endothelial cells. RA treatment resulted in increased levels of Nrf2 in ER stress-induced endothelial cells as assessed by qPCR and western blotting (Figures 3(a) and 3(b)). Further, RA-mediated Nrf2 activation was confirmed by Nrf2 translocation from cytosol to the nucleus. The RA-treated cells showed decreased levels of Nrf2 in cytosol fraction meanwhile increased levels in the nuclear fraction as demonstrated by western blotting. Further, the accumulation of cytosolic Nrf2 with a concomitant decrease in nuclear Nrf2 was observed in the TPG-treated endothelial cells was reversed upon RA pretreatment (Figure 3(c)). This confirms the role of RA-mediated Nrf2 activation against ER stress-induced ED.

### 3.4. Efficacy of RA on Nrf2 Activation and Its Downstream Regulation in ER Stress-Stimulated Endothelial Cells

**Figure 4:**
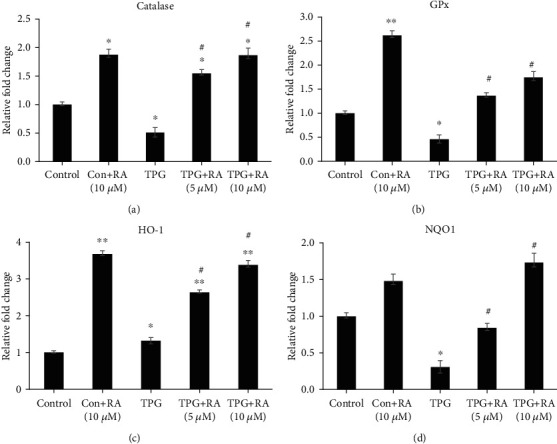
Effect of Rosolic acid on Nrf2 downstream targets such as (a) catalase (CAT), (b) glutathione peroxidase (GPx), (c) heme oxygenase-1 (HO-1), and (d) NAD(P)H: quinone-oxidoreductase-1 (NQO-1) expression in ER stress-induced EA.hy926 cells by qRT-PCR analysis. Data are represented as mean ± SD of three independent experiments. ∗Significant compared with Control group. ^#^Significant compared with TPG group; *P* < 0.05 determined by one-way ANOVA followed by Tukey's post hoc test (0, control; TPG, thapsigargin; 2.5, 5, 10, and 25, concentrations of Rosolic acid in *μ*M).

To study the effects of RA on Nrf2 downstream target genes, GPx, CAT, HO-1, and NQO1 expressions were studied. Upon treatment with TPG, the expressions of GPx, CAT, and NQO1 were downregulated, and HO-1 was marginally upregulated. Pretreatment with RA (5 and 10 *μ*M) effectively upregulated the expression of all the above-mentioned Nrf2 downstream genes in a dose-respective manner (Figures 4(a)–4(d)). RA treatment alone increased the expression of antioxidant genes, showing its potential antioxidant property.

Further, to validate the potential of RA on reducing the oxidative stress via Nrf2 signaling, we have determined the enzyme activities of SOD, CAT, and GPx in endothelial cells. The activity of SOD in endothelial cells with TPG-induced ER stress was found to be significantly decreased (0.71 units/mg protein), which was restored upon pretreatment with 10 *μ*M RA (1.12 units/mg protein) to near-normal levels (1.21 units/mg protein). A similar pattern of results was observed for both CAT and GPx activities with a significant reduction of enzyme activity in the TPG-treated endothelial cells with enzyme activity 30.19 *μ*mol/min/mg protein and 123.11 *μ*mol/min/mg protein, respectively. RA pretreatment dose-dependently showed improvement in CAT and GPx activities in endothelial cells. The quantitative results proved that RA also protected ER stress-induced endothelial cells against oxidative damage (Table 2).

### 3.5. Efficacy of RA against ER Stress in Endothelial Cells Assessed by ER Stress Markers

**Figure 5:**
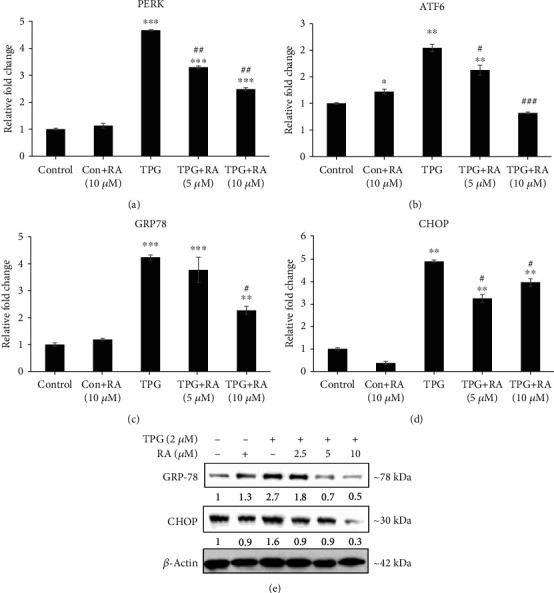
Effect of Rosolic acid against ER stress as assessed by ER stress markers such as (a) protein kinase R- (PKR-) like endoplasmic reticulum kinase (PERK), (b) activating transcription factor- (ATF-) 6, (c) glucose-regulated protein (GRP) 78, and (d) C/ERB homologous protein (CHOP) expression in ER stress-induced EA.hy926 cells qRT-PCR analysis. (e) The effect of Rosolic acid on GRP-78 and CHOP by western blotting in EA.hy926 cells under ER stress. Data are represented as mean ± SD of three independent experiments. ∗Significant compared with the control group. ^#^Significant compared with TPG group; *P* < 0.05 determined by one-way ANOVA followed by Tukey's post hoc test.

The protective effect of RA against TPG-induced ER stress in endothelial cells was evaluated by studying the expression of ER stress markers PERK, ATF-6, GRP78, and CHOP. All the tested ER stress gene expression was upregulated in TPG-treated cells, which confirm the stimulation of ER stress. Interestingly, pretreatment with RA (5 and 10 *μ*M) exhibited a dose-respective reduction in the PERK, ATF-6, and GRP78 expression but not CHOP expression (Figures 5(a)–5(d)). RA treatment alone did not show any significant variation in the level of ER stress gene expressions even after 24 h exposure in endothelial cells. Further, RA-mediated protection against ER stress was confirmed by the levels of the decreased ER chaperone GRP-78 by western blotting (Figure 5(e)).

### 3.6. Effect of RA against ROS Formation against ER Stress in Endothelial Cells as Assessed by DCFDA Assay Using Flow Cytometry

**Figure 6:**
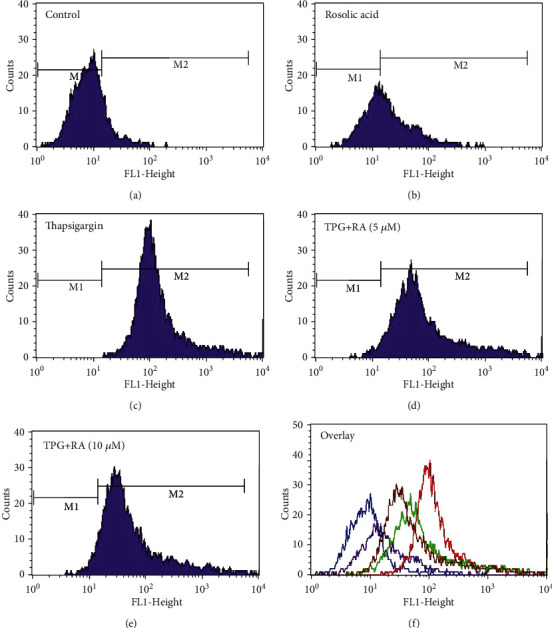
Analysis of reactive oxygen species formation in ER stress-induced EA.hy926 cells measured by flow cytometry using H2-DCFDA assay. Cells were pretreated with 5 and 10 *μ*g for 24 hours and then treated with thapsigargin for 2 hours, and then the cells were incubated with 25 *μ*M of DCFDA solution for 15 min and then harvested. After washing, the cells were subjected to flow cytometric analysis.

Intracellular ROS sensitive fluorescent probe DCFDA was used to evaluate whether RA could inhibit TPG-induced intracellular ROS generation in endothelial cells. As seen in Figure 6, there was a right shift in DCF peak in TPG- (Figure 6(c)) treated endothelial cells compared to control (Figure 6(a)), which confirm the increased free radical generation. Interestingly, a dose-dependent shift towards left was observed in the group pretreated with 5 (Figure 6(d)) and 10 (Figure 6(e)) *μ*M RA, respectively, proving the effect of RA in reducing intracellular ROS generation and thus protecting the endothelial cells against oxidative stress.

### 3.7. Antiapoptotic Efficacy of RA against ER Stress-Stimulated Endothelial Apoptosis

**Figure 7:**
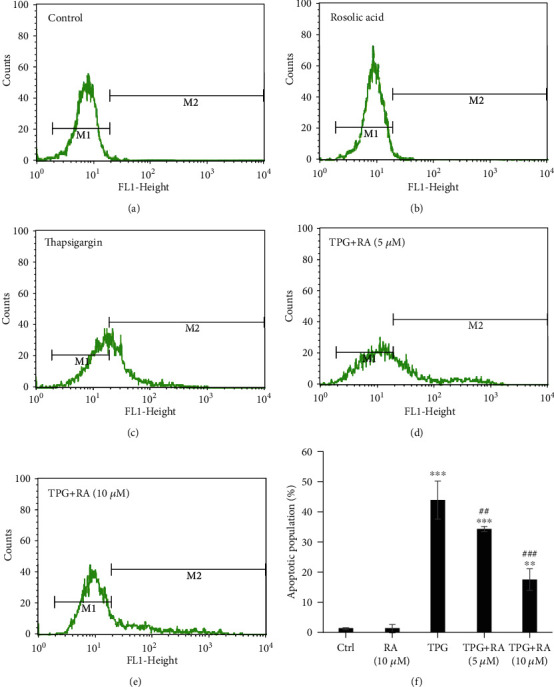
Analysis of apoptotic frequencies. Rosolic acid-treated EA.hy926 cells under ER stress was measured by flow cytometry using the Annexin-V labelling assay. Cells were pretreated with 5 and 10 *μ*g for 24 hours and then treated with Thapsigargin for 2 hours and then incubated with FITC-labelled Annexin-V. After washing, the cells were subjected to flow cytometric analysis. The intensity of the Annexin-V-Fluos signal is represented on the *x*-axis. A significant peak shift towards right was observed in the endothelial cells exposed to TPG (c) in comparison with the untreated cells (a) whereas a dose-dependent shift was observed towards the control level in the RA-pretreated endothelial cells (d, e). No peak shift was observed in the cells exposed only RA (b). The apoptotic population (%) against the effect of Rosolic acid against the TPG-induced ER stress in EA.hy926 cells (f).

Annexin-V-FITC labeling assay by flow cytometry were implemented to examine the antiapoptotic property of RA against TPG-induced ER stress in endothelial cells. The apoptotic cell population was elevated by 43.87% (Figure 7) in TPG-treated cells. A dose-dependent reduction by 34.28% and 17.49% was observed in 5 and 10 *μ*M on RA pretreatment, respectively. There was no significant change seen in the cell population treated with RA alone compared with untreated EA.hy926 cells. This shows that the RA protects endothelial cells against apoptosis induced by ER stress.

### 3.8. Development of Nrf2-Knockout Endothelial Cells Using CRISPR/Cas9

**Figure 8:**
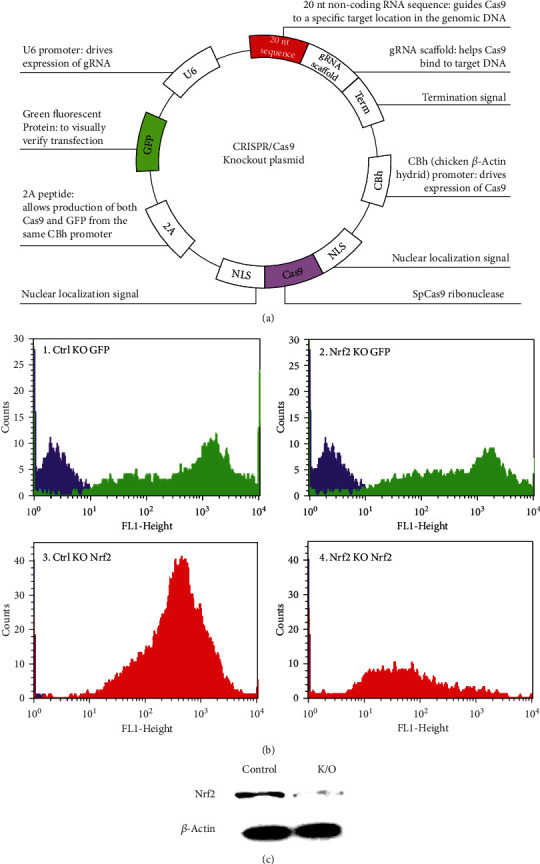
CRISPR/Cas9-mediated knockout of Nrf2 expression in EA.hy926 cells. The endothelial cells were transfected with Nrf2-CRISPR (a). CAS9-GFP plasmid and GFP+ cells expression were screened using FACS (b). Western blot analysis for Nrf2-expression was performed to confirm successful Nrf2-depletion (c).

To determine whether the protective efficacy of RA was facilitated via activation of Nrf2, we have developed Nrf2-knockout endothelial cells using CRISPR/Cas9. The knockout cells were subjected to flow cytometry analysis, and a comparable level of GFP expression was recorded in both control and Nrf2 KO EA.hy926 cells, which shows equal transfection efficiency of both the plasmids. However, Nrf2 expression was completely lost in Nrf2 KO plasmid-transfected EA.hy926 cells when compared to the scrambled transfected control cells, as seen in Figure 8. Further, this result was validated using western blot analysis, where a complete reduction in the Nrf2 level was recorded in KO cells, and those cells were used for the further mechanistic study.

### 3.9. Efficacy of RA on TPG-Stimulated ER Stress in Nrf2 KO Endothelial Cells

**Figure 9:**
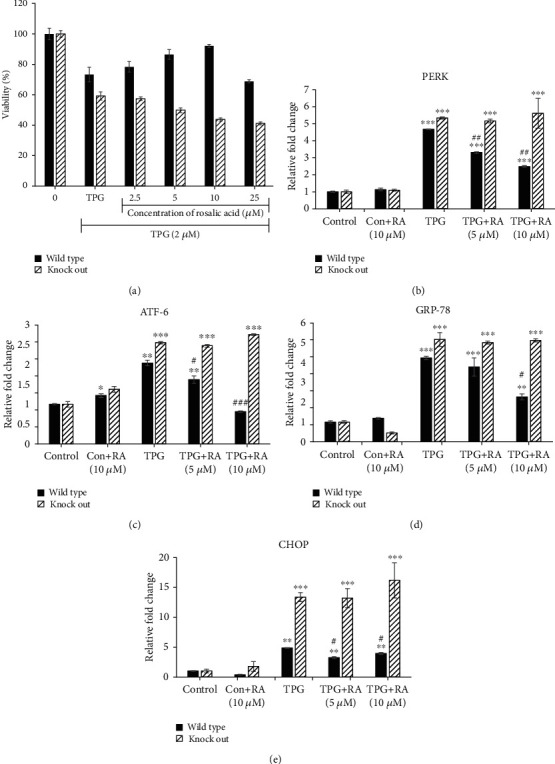
Effect of Rosolic acid on ER stress-induced toxicity in Nrf2 KO and wild-type EA.hy926 cells. (a) Cytoprotective effect of Rosolic acid in wild-type and Nrf2 KO EA.hy926 cells against thapsigargin-induced toxicity as assessed by MTT assay. (b) Effect of Rosolic acid on ER stress-induced toxicity in Nrf2 KO and wild-type cells as assessed by ER stress markers, (b) protein kinase R- (PKR-) like endoplasmic reticulum kinase (PERK), (c) activating transcription factor- (ATF-) 6, (d) glucose-regulated protein (GRP) 78, and (e) C/ERB homologous protein (CHOP) gene expression levels in ER stress-induced in wild-type and Nrf2 KO EA.hy926 cells using thapsigargin by qRT-PCR analysis. Data are represented as mean ± SD of three independent experiments. ∗Significant compared with the control group. ^#^Significant compared with TPG group; *P* < 0.05 determined by one-way ANOVA followed by Tukey's post hoc test.

MTT assay was performed to check the modulation in the cytoprotective effect of RA on TPG- induced ER stress in Nrf2 KO cells. Increased sensitivity to apoptosis upon ER stress was observed in Nrf2 KO cells, which was confirmed by a 59.2% reduction in the cell viability upon exposure to TPG. Further pretreatment with different concentrations of RA (2.5–25 *μ*M) for 24 h failed to protect the cells against ER stress (Figure 9(a)). This confirms that RA protects the endothelial cells against ER stress through the stimulation of the Nrf2 signaling pathway.

### 3.10. Effect of RA on ER Stress Markers in Nrf2-Knockout Endothelial Cells

Further, we compared the effect of RA on the ER stress marker expressions, PERK, ATF-6, GRP-78, and CHOP in both Nrf2 wild-type and Nrf2 KO cells subjected to ER stress. RA-pretreated cells exhibited augmented ER stress marker expressions when compared to control cells. RA-mediated protection against ER stress was not observed in Nrf2 KO cells unlike in wild-type EA.hy926 cells where the protection was evident (Figure 9(b)).

### 3.11. Molecular Mechanism of RA-Mediated Protection against ER Stress through Proteomic Analysis

**Figure 10:**
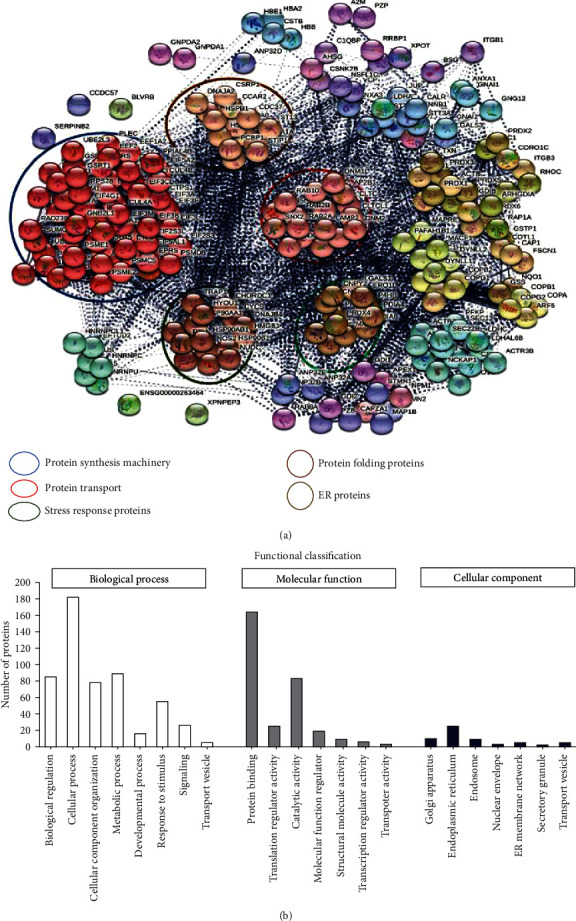
(a) Visualization of protein–protein interaction network of differentially regulated proteins using STRING 10.5 web tool with “MCL inflation parameter (3.0) and medium confidence (0.4).” The colour of nodes corresponds to a cluster, and intercluster edges are represented by dashed lines. MCL, Markov clustering algorithm; STRING, Search Tool for the Retrieval of Interacting Genes. (b) Bar chart showing biological process, molecular function and cellular localization of differentially regulated proteins using the PANTHER software. The total number of proteins in each division is shown on the *y*-axis.

To study the molecular mechanism using proteomic analysis using LC-MS/MS on a Q Exactive mass spectrometer, we have taken three groups, including control (Cont), TPG-treated endothelial cells (TPG), and RA-treated TPG-induced endothelial cells (TPG+RA). The results revealed that among the identified 1370 proteins, 35.03% of the proteins were known with one peptide, 18.61% with two peptides, 10.58% with three peptides, 6.27% with four peptides, and 27% of proteins with more than five peptides. The proteins with two or more peptides were considered for further analysis. We found 296 proteins were differentially expressed with twofold or more change and two or more peptides in TPG-treated cells when compared to the control in which 143 downregulated and 153 upregulated. The STRING 11 online web tool was used to generate protein-protein interaction networks of differentially regulated proteins, with the settings of “MCL inflation parameter (3.0) and medium confidence (0.4)” (Figure 10(a)). We have identified eight major protein interaction network clusters, which are mainly involved in protein synthesis machinery, protein transport, stress response proteins, protein-folding proteins, and ER proteins.

The cellular organelle localization of the differentially regulated proteins was identified as 39.3% from the nucleus, 13.1% from the endoplasmic reticulum, 6.6% from the mitochondria, 8.2% from the Golgi apparatus, and 30.3% from the vacuoles using PANTHER. Further, the class of differentially regulated proteins was found that 16.9% proteins were associated with cytoskeletal proteins, 15.1% with translational proteins, 9.6% with membrane traffic proteins, 8.4% with chaperones, and 14.5% in protein modifying enzymes (Figure 10(b)).

Further, the differentially regulated proteins were subjected to DAVID for their annotation. We found a total of 17 clusters with an enrichment score of more than 3. We have considered the clusters with an enrichment score of more than 10. The top three cellular components are occupied by “cell-cell adhesion proteins” with an enrichment score of 17.2. “Cell Chaperone” has an enrichment score of 13.35, and “GTP-binding” has an enrichment score of 10.88 (Table 3).

The list of DAVID–KEGG pathways of differentially regulated proteins are depicted in Table 4. 10.6% of the proteins belong to protein processing in the endoplasmic reticulum. The other classes of proteins include RNA transport, proteasome, antigen processing and presentation, phagosome, estrogen signaling pathway, leukocyte transendothelial migration, adherent junction, and AMPK signaling pathway.

## 4. Discussion

Though several approaches have been established to improve the endothelial functions, drugs that can effectively treat microvascular complications are limited. This study was performed to determine RA, a polyphenol isolated from the rhizome of *Plantago asiatica* L. which can be used as a therapeutic agent to treat hyperglycemia-induced ER stress and endothelial dysfunction. We provided the first line of evidence that RA attenuates ER stress-induced endothelial apoptosis. Further, we also provide evidence that attenuation of ER stress and apoptosis is a result of the activation of Nrf2 and its signaling network.

Generally, endothelial cells are metabolically dynamic, contributing to various physiological mechanisms, including maintenance of blood fluidity, permeability, angiogenesis, and the control of vasomotor tone [34]. Oxidative stress, cytokines, and ER stress often cause endothelial cell injury, which disrupts ER homeostasis ensuing the accretion of unfolded or misfolded proteins, and thus activation of an adaptive response called UPR. The main function of UPR is to decrease ER stress and prolong cell endurance. If ER stress is extended, the signaling shifts from prosurvival to prodeath, which leads to ER stress-stimulated apoptosis [35, 36]. The ER function disruption has often been connected to the progression of numerous illnesses, including diabetes [37–39]. Although the causative role is yet to be established, the underlying mechanisms by which ER dysfunction can recruit or stimulate a disease state have been suggested [40]. Few investigations have established that ER stress promotes apoptosis through the caspase activation in endothelial cells [41, 42]. Earlier investigations have also revealed that endothelial cells are highly susceptible to ER and oxidative stress, a pathological cause of endothelial injury [43]. Besides, a rate-limiting enzyme of the hexosamine pathway, fructose-6 phosphate amidotransferase, is overexpressed in diabetic settings, which contributes ER stress through inducing UPR-related gene expressions that eventually recruit proinflammatory and apoptosis mechanisms [44]. Also, increased levels of ER stress markers, CHOP, GRP-78, PERK, and ATF-6 are seen in cells under ER stress [45]. Mozzini et al. revealed high levels of GRP-78 and CHOP in the PBMCs of chronic T2DM patients to be linked to the loss of Nrf2/ARE defense [46]. The ER and oxidative stress responses were correlated to PERK-mediated activation of ATF4 and Nrf2 [47]. Yang et al. demonstrated the effects of palmitic acid toxicity on apoptosis of H9c2 cardiomyocytes, uncovered the corelation between ER and oxidative stress apoptotic signaling pathways, and further showed the ameliorative effect of N-acetylcysteine against oxidative and ER stress-induced cell apoptosis [48]. Recently, ER stress has been shown to have a direct link with the inflammatory response pathway. Lei et al. showed that the administration of lipopolysaccharide results in the activation of ER stress as assessed by marker protein expression GRP78 and CHOP along with the increased levels of proinflammatory cytokines such as IL-1*β*, IL-6, IL-8, and TNF-*α* in mouse granulosa cells. Also, inhibition of ER stress by 4-phenylbutyrate (4-PBA) reduced the expression of proinflammatory cytokines and ER stress markers, CHOP and GRP78 [49].

A well-known ER stress inducer, thapsigargin, is an appropriate irreversible inhibitor of sarco/endoplasmic reticulum Ca^2+^-ATPase (SERCA) which induces an elevation of calcium levels and causes fragmentation of DNA, resulting in cell demise by triggering apoptotic mechanisms or alteration in architecture of the cell [50]. In the current investigation, the two ER stress markers CHOP and GRP78 were induced in thapsigargin-exposed endothelial cells.

Rosolic acid is known for its beneficial effects on the cardiovascular system [28]. RA-induced HO-1 in endothelial cells thereby showed antioxidant, anti-inflammatory, and antiapoptotic effects in several pathophysiological mechanisms stimulated cardiac damage [28]. In the current study, we indicated that RA defends endothelial cells against apoptosis and ER stress, ensuring a favorable effect on the vascular system. The underlying mechanisms explaining the function of RA in endothelial cells and their regulation of physiological processes have not been explored yet. This is the opening line of proof highlighting the role of RA-mediated Nrf2 activation in endothelial cells. Nrf2 is a chief regulator of the cellular stress response, and the activation of Nrf2 by small molecules has shown protective effects against the development of ED both *in vitro* and *in vivo* studies [16]. In the present study, RA activated Nrf2, thereby triggering its downstream regulations, including HO-1, NQO1, GPx, and CAT. Further, we have used GST-ARE-Luc and hNQO1-ARE-Luc reporter gene constructs to confirm the RA-mediated Nrf2 activation potential of RA in EA.hy926 cells and recorded a dose-dependent increase in the Nrf2 expression levels. In agreement with the report published by Foresti et al., RA dose-dependently increased the expression of HO-1 [28]. The Nrf2 translocation from cytosol to nucleus further conformed the Nrf2 activation potential of RA as demonstrated by western blot.

Our data demonstrated TPG exposure decreased expression of HO-1, NQO1, GPx, and CAT, and a dose-dependent increase was observed in the RA pretreatment. Also, the activities of SOD, CAT, and GPx have further confirmed the role of RA in Nrf2 mediated cytoprotective action against oxidative stress in endothelial cells. It is well known that Nrf2 is a master regulator of detoxifying enzymes exerting the effect through its different downstream pathways targeting HO-1, NQO1, GPx, and CAT. Maamoun et al. have already proved that HO-1 independently shows a protective effect in HUVECs against ER stress-mediated endothelial apoptosis and also impaired angiogenesis in a high-lucose environment [51]. Along with HO-1, Chen et al. reported that Nrf2 downstream targets like GPx and glutathione (GSH) showed protection of endothelial cells against H_2_O_2_-induced cytotoxicity [52]. It was evidenced that under persistent high glucose and more prominently in oscillating high glucose conditions, there was a high increase in the apoptotic population of Primary Human Coronary Artery Endothelial Cells (HCAEC) accompanied by the suppressed expression of Nrf2 and HO-1 [53]. NQO1 and HO-1 have also been implicated in promoting angiogenesis which are reported in diabetic wound healing in various *in vitro* and *in vivo* models [54]. The role of SOD, CAT, and GPx against oxidative stress has been demonstrated in many vascular complications [55, 56]. Many studies have reported the association of reduced SOD activity and increased vascular oxidative stress and also has been implicated in the vascular disorders with ED as the underlying cause [57–60]. Our earlier studies confirmed that pterostilbene, an Nrf2 activator, conferred protection against oxidative stress by elevating the levels of Nrf2 and its downstream genes and against cytokine stress [22, 24]. Further, the proteomic profiling of pancreatic tissue isolated from pterostilbene treated mice demonstrated that the majority of differentially regulated proteins were associated with oxidative and ER stress pathways [61].

In the current study, we observed a greater Nrf2 expression and downstream targets and significant inhibition in the expression of PERK, CHOP, and GRP78 in RA-treated endothelial cells. Our outcomes show that the cytoprotective effect of RA on endothelial apoptosis was mediated by the activation of Nrf2.

To determine whether the protective efficacy of RA was facilitated via activation of Nrf2, we have developed Nrf2 knockout endothelial cells using CRISPR/Cas9. We found a very minimal expression of Nrf2 in knockout cells. The probable reason could be the biological plasticity which rescues target Nrf2 activity in CRISPR knockouts. This concept has been observed in three truncated targets, BRD4, DNMT1, and NGLY1, which revealed partial preservation of protein function even after knock out by CRISPR/Cas9{Smits, 2019 #2260}. The Nrf2 cytoprotective adaptive response has evolved to be a powerful protective strategy for organisms against exposure to various insults. This Nrf2 signaling can provide plasticity in the cellular response to a wide array of chemical agents.

Since RA failed to show the protective effect against ER stress in Nrf2 KO by CRISPR/Cas9 in this study, it cannot be ruled out that the increased expression of Nrf2 caused inhibition of ER stress markers such as GRP78 and CHOP, thus restoring ER homeostasis. Additional investigations are needed to extrapolate the function of RA in ER stress-stimulated apoptosis in animal models.

There are many studies that have linked decreased Nrf2 activity leads to increased oxidative damage, leading to many pathophysiological disorders, including obesity, diabetes mellitus, and atherosclerosis which are the major contributors of cardiovascular disorders [62]. Few other Nrf2 activators have also shown promising therapeutics against hyperglycemia and ED as demonstrated by Wang et al. Cinnamaldehyde, an Nrf2 activator, preserved nitric oxide (NO) levels and upregulated Nrf2 levels and its downstream regulations in the HUVEC cells and mouse aortas in the hyperglycaemic environment [63]. Another major Nrf2 activator, dh404, upregulated Nrf2 in Akita mice and diabetic human aortic endothelial cells (HAECs) and proved to be a potential therapeutic agent against diabetes-induced ED [17]. The Nrf2 activators such as curcumin, resveratrol, and sulforaphane have also been explored as adjuvants against diabetic complications [64–66]. Ding proved that the dietary ellagic acid showed a protective effect against oxidative stress developed ED and atherosclerosis through the Nrf2 activation pathway in human umbilical vein endothelial cells and diabetic mice [67].

In our results, we found that RA has a protective effect against ER stress and that there are concentration-dependent effects. Furthermore, we used LC-MS/MS to evaluate the molecular mechanism of endothelial cells. By analyzing the differentially regulated proteins in RA-pretreated endothelial cells exposed to ER stress, we found that the differentially regulated proteins were mainly enriched for proteins from the protein-folding machinery and cell-cell adhesion proteins. It has been reported that under pathophysiological stimuli, the ER homeostasis has been disrupted and leads to the accumulation of misfolded and or unfolded proteins in the ER until correctly folded or demolished [68]. Sustained ER stress has been associated with ED. In the present study, treatment of endothelial cells with RA improved its function by inhibiting ER stress through the regulation of protein-folding machinery.

The adenosine monophosphate-activated protein kinase (AMPK) is a central protein kinase that has been reported to enhance the various protecting cells under different stress stimuli such as hypoxia [69], glucose deprivation [70], and hypertrophy [71] and act as a potential target for resisting atherosclerosis. It has been reported that the key target cells in cardiovascular endothelial cells or smooth muscle cells are directly or indirectly controlled by AMPK [72]. Few reports demonstrated that activation of AMPK is connected with protein synthesis inhibition through elongation factor 2 (eEF2) phosphorylation in heart muscles [73, 74]. Terai et al. reported that AMPK possibly inhibits the signaling of ER-linked apoptosis in heart muscles [73]. Zimmermann et al. demonstrated AMPK employs a positive effect in mouse embryonic fibroblast-associated signaling of Nrf2/heme oxygenase- (HO-) 1 [75]. Numerous studies suggested that the collaboration of Nrf2 and AMPK contributes to the progressive signaling networks [76, 77]. The AMPK activation has been found to stimulate the unfolded protein response and reduce ER stress in many stressors [78]. Metformin, a known stimulator of AMPK inhibits GRP78 and phosphorylates eukaryotic initiation factor-2*α* resulting in a reduction of THP-induced ER stress [78]. The silencing of NRF2 during oxidative stress ensuing a constant activation of AMPK contributes to hyperactivation of autophagy [79]. Our results revealed that the RA-facilitated defense against ER stress might be through the amelioration of the AMPK pathway. In addition to these pathways, other significantly changed pathways were cell-cell adhesion proteins, GTP-binding proteins, stress response proteins, proteasomal pathway, RNA transport, etc. To our understanding, this is the preliminary pilot study that validates that RA exerts a direct impact on alleviating ER stress-mediated alterations in defending endothelial cells.

## 5. Conclusions

In conclusion, Rosolic acid is a potent Nrf2 activator and is protective against ER stress-induced endothelial apoptosis. This protective effect of Rosolic acid is attributed to the induction of Nrf2 and can be developed into a possible therapeutic intervention against ED in diabetes and related cardiovascular diseases. Further *in vivo* studies are essential to confirm the effect of Rosolic acid in diabetes.

## Figures and Tables

**Table 1:** List of primers and its sequences used for the study.

S. No.	Gene	Forward	Reverse
1	NRF2	TGTAGATGACAATGAGGTTTC	ACTGAGCCTGATTAGTAGCAA
2	GPx	TTCCCGTGCAACCAGTTTG	TTCACCTCGCACTTCTCGAA
3	SOD	GAAGGTGTGGGGAAGCATTA	ACATTGCCCAAGTCTCCAAC
4	NQO1	AGGATGGAAGAAACGCCTGG	TCAGTTGGGATGGACTTGCC
5	HO1	GGGAATTCTCTTGGCTGGCT	AACTGAGGATGCTGAAGGGC
6	GRP78	GCGTCGGCGTGTTCAAGA	CAGACGGGTCATTCCACGTG
7	PERK	GAACCAGACGATGAGACA GAG	GGATGACACCAAGGAACCG
8	CHOP	GTACCTATGTTTCACCTCCTG G	TGGAATCTGGAGAGTGAGGG
9	ATF 6	CGGAGCCACTGAAGGAAG ATA	TTGAGTCTTGGGTGCTGCTG
10	GAPDH	AAGAAGGTGGTGAAGCAGGC	GTCAAAGGTGGAGGAGTGGG

**Table 2:** Effect of Rosolic acid on activities of enzymatic antioxidants in ER stress-induced EA.hy926 cells.

Groups	SOD (units/mg protein)	CAT (*μ*mol/min/mg protein)	GPx (*μ*mol/min/mg protein)
Control	1.21 ± 0.04	39.62 ± 0.8	150.51 ± 9.61
RA (10 *μ*M)	1.43 ± 0.09∗∗	42.21 ± 4.4	192.04 ± 13.72∗∗∗
TPG (2 *μ*M)	0.71 ± 0.21∗∗	30.19 ± 1.1∗∗∗	123.11 ± 5.69∗∗∗
RA (5 *μ*M)+TPG (2 *μ*M)	0.81 ± 0.12∗∗∗	31.62 ± 2.7∗∗∗	118.06 ± 7.78∗∗∗^###^
RA (10 *μ*M)+TPG (2 *μ*M)	1.12 ± 0.16^##^	33.41 ± 0.2∗∗∗^###^	135.59 ± 9.89∗∗^###^

Data are represented as mean ± SD of three independent experiments. ∗Significant compared with the control group. ^#^Significant compared with TPG group; *P* < 0.05 determined by one-way ANOVA followed by Tukey's post hoc test.

**Table 3:** List of DAVID annotation clusters with enrichment score above 10.

Enrichment score: 17.2							
Category	Term	Count	*P* value	Fold enrichment	Bonferroni	Benjamini	FDR

Annotation cluster 1							
GOTERM_MF_DIRECT	Cadherin binding involved in cell-cell adhesion	33	2.1E-19	7.9	9.1E-17	3.0E-17	3.0E-16
GOTERM_CC_DIRECT	Cell-cell adherens junction	33	5.3E-19	7.7	2.1E-16	3.0E-17	7.4E-16
GOTERM_BP_DIRECT	Cell-cell adhesion	28	2.2E-15	7.2	3.5E-12	1.8E-12	3.7E-12
Enrichment score: 13.35							
Annotation cluster 2							
UP_KEYWORDS	Chaperone	32	1.4E-25	13.3	3.6E-23	1.2E-23	1.8E-22
GOTERM_MF_DIRECT	Unfolded protein binding	26	3.1E-23	16.4	1.3E-20	6.5E-21	4.3E-20
GOTERM_BP_DIRECT	Protein folding	30	2.4E-22	11.6	3.8E-19	3.8E-19	4.0E-19
UP_KEYWORDS	Stress response	22	1.4E-20	18.4	3.6E-18	6.0E-19	1.8E-17
INTERPRO	Heat shock protein Hsp90	10	5.4E-16	68.9	2.7E-13	2.7E-13	8.0E-13
INTERPRO	Heat shock protein Hsp90, N-terminal	8	2.2E-12	67.3	1.1E-9	5.3E-10	3.1E-9
INTERPRO	Ribosomal protein S5 domain 2-type fold	12	8.5E-12	20.7	4.1E-9	6.8E-10	1.2E-8
GOTERM_BP_DIRECT	Response to stress	12	9.4E-10	13.7	1.5E-6	1.4E-7	1.6E-6
INTERPRO	Histidine kinase-like ATPase, ATP-binding domain	9	8.3E-9	20.7	4.0E-6	3.3E-7	1.2E-5
SMART	HATPase_c	6	8.9E-8	46.1	8.5E-6	8.5E-6	9.8E-5
INTERPRO	Heat shock protein Hsp90, conserved site	4	8.9E-6	75.8	4.3E-3	2.2E-4	1.3E-2
PIR_SUPERFAMILY	Heat shock protein, HSP90/HTPG types	4	7.9E-5	35.2	2.4E-3	2.4E-3	6.7E-2
Enrichment score: 10.88							
Annotation cluster 3							
UP_KEYWORDS	GTP-binding	30	1.1E-16	7.3	2.9E-14	4.1E-15	1.4E-13
UP_SEQ_FEATURE	Nucleotide phosphate-binding region: GTP	28	1.1E-15	7.4	8.7E-13	8.7E-13	1.7E-12
UP_KEYWORDS	Nucleotide-binding	64	2.0E-15	3.0	5.1E-13	6.4E-14	2.6E-12
GOTERM_MF_DIRECT	GTPase activity	26	5.4E-15	7.7	2.3E-12	4.5E-13	7.5E-12
GOTERM_MF_DIRECT	GTP binding	32	6.5E-15	5.8	2.7E-12	4.6E-13	9.1E-12
INTERPRO	Small GTP-binding protein domain	19	8.0E-12	8.6	3.8E-9	7.6E-10	1.1E-8
UP_KEYWORDS	Prenylation	18	1.9E-11	9.0	4.8E-9	4.3E-10	2.4E-8
UP_SEQ_FEATURE	Lipid moiety-binding region: S-geranylgeranyl cysteine	15	5.1E-11	11.4	4.0E-8	2.0E-8	7.8E-8
UP_SEQ_FEATURE	Short sequence motif: effector region	14	2.8E-10	11.4	2.2E-7	7.3E-8	4.3E-7
INTERPRO	P-loop containing nucleoside triphosphate hydrolase	38	3.5E-10	3.3	1.7E-7	2.1E-8	5.0E-7
INTERPRO	Small GTPase superfamily	15	4.3E-9	8.2	2.1E-6	1.9E-7	6.2E-6
GOTERM_BP_DIRECT	Small GTPase-mediated signal transduction	19	1.7E-8	5.4	2.8E-5	1.7E-6	2.9E-5
GOTERM_MF_DIRECT	GDP binding	9	9.7E-7	11.5	4.1E-4	3.4E-5	1.4E-3
UP_KEYWORDS	Lipoprotein	27	1.1E-5	2.7	2.9E-3	1.1E-4	1.5E-2

**Table 4:** List of DAVID–KEGG pathways of differentially regulated proteins.

Term	Count	%	*P* value	Fold enrichment	Bonferroni	Benjamini	FDR
Protein processing in endoplasmic reticulum	26	10.6	3.6E-14	6.7	6.0E-12	6.0E-12	4.3E-11
Endocytosis	20	8.1	1.8E-6	3.6	3.1E-4	7.8E-5	2.2E-3
RNA transport	17	6.9	1.5E-6	4.3	2.5E-4	8.3E-5	1.8E-3
Proteasome	9	3.7	5.2E-6	9.0	8.7E-4	1.8E-4	6.3E-3
Antigen processing and presentation	9	3.7	3.0E-4	5.2	4.9E-2	8.4E-3	3.6E-1
Phagosome	12	4.9	5.7E-4	3.5	9.1E-2	1.1E-2	6.9E-1
Estrogen signaling pathway	9	3.7	1.7E-3	4.0	2.6E-1	2.9E-2	2.1E0
Leukocyte transendothelial migration	9	3.7	4.4E-3	3.4	5.3E-1	6.6E-2	5.3E0
Adherent junction	6	2.4	2.2E-2	3.7	9.8E-1	1.9E-1	2.4E1
AMPK signaling pathway	8	3.3	2.1E-2	2.8	9.7E-1	1.9E-1	2.3E1
Rap1 signaling pathway	11	4.5	2.1E-2	2.3	9.7E-1	2.0E-1	2.3E1
Regulation of actin cytoskeleton	10	4.1	4.9E-2	2.1	1.0E0	3.5E-1	4.6E1
Pathways in cancer	15	6.1	6.2E-2	1.7	1.0E0	3.5E-1	5.4E1
Sphingolipid signaling pathway	7	2.8	5.5E-2	2.6	1.0E0	3.5E-1	5.0E1
Pyruvate metabolism	4	1.6	6.1E-2	4.4	1.0E0	3.6E-1	5.4E1
Cysteine and methionine metabolism	4	1.6	5.4E-2	4.6	1.0E0	3.6E-1	4.9E1
Platelet activation	7	2.8	7.5E-2	2.4	1.0E0	3.8E-1	6.1E1
Endocrine and other factor-regulated calcium reabsorption	4	1.6	8.1E-2	3.9	1.0E0	3.9E-1	6.4E1
Nucleotide excision repair	4	1.6	9.0E-2	3.7	1.0E0	4.0E-1	6.8E1

## Data Availability

The complete data of the findings of this study are available in “Results” and also from the corresponding author.

## Supplementary Materials

Supplementary Materials Figure S1: description of detailed methodology of proteomics.
