# Supplementary material for: Pharmacological Activation of Nrf2 by Rosolic Acid Attenuates Endoplasmic Reticulum Stress in Endothelial Cells
Source: Oxid Med Cell Longev. 2021 Apr 8;2021:2732435. doi: 10.1155/2021/2732435 (PMC8052152; doi:10.1155/2021/2732435)
Supplement: Supplementary Materials — Figure S1: description of detailed methodology of proteomics. [file 2732435.f1.pdf]

Description of supplementary material

Description of detailed methodology of proteomics

Proteomic investigations performed by nano LC-MS/MS analysis

Protein extraction and precipitation from the endothelial cells: The protein samples for proteome analysis (LC-MS/MS) were prepared from the endothelial cells (n=3/group) by homogenizing using RIPA buffer supplemented with protease inhibitor cocktail. The lysate were centrifuged at 12,000 x g at 4°C for 15 min and the supernatant was taken in a separate tube and mixed with 4 volumes of ice-cold 80% acetone and placed at -20°C for overnight. The protein precipitate was collected by centrifugation at 12,000 x g at 4°C for 15 min and the resultant pellet was washed with 1 mL of ice-cold 80% acetone. The final protein precipitate was collected by centrifugation at 12,000 x g at 4°C for 15 min. A portion of the protein precipitate was dissolved in 20-30 µL of the RapiGestSF surfactant (Waters Corporation, Milford, USA) and quantified fluorimetrically using the Qubit Protein assay kit (Molecular Probes, Life Technologies, Oregon, USA). The protein solution was stored at -80°C until further analysis.

Reduction/alkylation: The protein samples (10 µg) were reduced with DTT, (dithiothreitol 5 mM, 30 min, 60°C), after cooling alkylated with iodoacetamide (15 mM, 30 min, at room temperature)Subsequently, digested with modified trypsin (1:50 w/w) (Promega, Medison, WI, USA) for 24 h at 37°C. The trypsin activity was arrested by adding TFA (0.5% 30 min, 37°C) for. The hydrolysed detergents and undigested protein, if any, are removed by centrifuging the tubes at 12,000 x g for 10 min, at 4°C.

Desalting of the digested proteins: The sample aliquot containing 5 µg of initial protein was desalted using µ-C18-ZipTips (Waters Corporation, Milford, USA) according to the manufacturer's instructions.

LC-MS/MS analysis Protein digests were analysed by nano flow high-performance liquid chromatography (HPLC)-electrospray tandem mass spectrometry (LC-MS/MS). A total volume of 10 µL of the protein digest (equivalent to 0.4µg of peptides) was injected onto a C18 capillary trapped column (0.3 mm x 5 mm, 5µm particle size, Chemicals Evaluation and Research Institute, Tokyo, Japan) and separation was performed on a C18 column (0.1 mm x 150 mm, 3 µm particle size, Zaplous, AMR Incorporated, Tokyo, Japan). A LC-MS/MS separation was performed using a nanoflow UHPLC (Advance LC, Bruker Corporation, Fremont, CA, USA) using 0.1% Formic Acid (A) in water and 100% ACN (B) at the flow rate of 400 nl/min. The gradient was then linearly increased from 2% of B to 40% over 100 min, and finally to 98% over 5 min and maintained at 98% B for 10 min then at 2% B. Peptides were

ionized in a captive ion source (Bruker Corporation, Fremont, CA, USA) and analysed by a voltage of 1.6kV in the positive ion mode and in data-dependent acquisition mode in the m/z range between 300 and 2000 at 75,000 resolutions using a hybrid Quadrupole-Orbitrap mass spectrometer (Q Exactive Thermo Fisher Scientific, San Jose, CA). The peptides with 10 high-intensity signals in the MS mode were fragmented with collision energy of 35 eV and analysed in Qtrap. To exclude ions with similar m/z and avoid interferences, the data-dependent mode was also set for a repeat duration of 10s.

MS Data analysis The MS fragmentation data from Quadrupole-Orbitrap were obtained using 'X calibur software' (version 2.0, Thermo Fisher Scientific, San Jose, CA) was used to control and to acquire. The raw MS and MS/MS spectra were imported to Progenesis QI (1.0, Nonlinear Dynamics, Waters Corporation, Milford, USA) for peptide identification and a 3D plot depicting retention time (RT), m/z and intensity of all the peptide ions was generated for all the samples. The intensities of each peptide ion signal in all the samples were normalized against each other using an automated normalization procedure and quantified. All sample features were aligned according to RT by placing manual vectors and by automatic alignment to maximally overlay all the two-dimensional (m/z and retention time) feature maps. Then, the single-charged peptides and the peptides with charge states higher than seven were excluded from the analysis.

The MS and MS/MS peak lists generated by the Progenesis LC–MS software were used for protein identification by Mascot2.4 (Matrix Science, UK) MS/MS ions search with Swissprot database 2013_10 with following setting: enzyme - trypsin and allow two missed cleavages; taxonomy –human; mass tolerance for parent MS - 10 ppm; . MSMS tolerance - 0.1mmu; and instrument - ESI-Trap. The search results stored in XML file format was imported into Progenesis for further data analysis.

For label free quantification, information of the identified peptides is transferred to Progenesis and peptides are assigned to respective proteins. After peptide and protein identification, the abundance of each peptide was calculated from all its constituent peptide ions. Then relative quantification of the proteins was performed using Hi-N peptides by comparing each single protein across all runs. Only the high abundant peptides (1E+05 and greater), scored more than 40, 2 and more hits in MS/MS spectra, and have no conflicts for the protein level assignments were used for quantification of the proteins. For quantification, the total cumulative abundance was calculated by summing the individual abundance of all peptides assigned to each protein.

Two sample t-test ($P<0.05$) was used for identifying the differentially expressed proteins in various test groups: TPG/Cont, and THP+RA/THP. Subsequently, the fold-change for the differentially expressed proteins were calculated by taking the ratio between the average values of treated and control groups. Using DAVID and PANTHER bioinformatics tools, Gene Ontology (GO) for localization, biological process and molecular function were predicted. Protein-protein interaction network analysis of the differentially expressed proteome was performed by using the STRING (Search Tool for the Retrieval of Interacting Genes) version 10.5 (http://string-db.org) database using "MCL inflation parameter (3.0) and medium confidence (0.4)".
